# Fitness trade-offs of group formation and movement by Thomson's gazelles in the Serengeti ecosystem

**DOI:** 10.1098/rstb.2017.0013

**Published:** 2018-03-26

**Authors:** John M. Fryxell, Andrew M. Berdahl

**Affiliations:** 1 Department of Integrative Biology, University of Guelph, Guelph, Ontario, Canada N1G 2W1; 2 Santa Fe Institute, Santa Fe, NM 87501, USA; 3 School of Aquatic and Fishery Sciences, University of Washington, Seattle, WA 98105, USA

**Keywords:** fission–fusion, fitness, foraging, model, predation

## Abstract

Collective behaviours contributing to patterns of group formation and coordinated movement are common across many ecosystems and taxa. Their ubiquity is presumably due to altering interactions between individuals and their predators, resources and physical environment in ways that enhance individual fitness. On the other hand, fitness costs are also often associated with group formation. Modifications to these interactions have the potential to dramatically impact population-level processes, such as trophic interactions or patterns of space use in relation to abiotic environmental variation. In a wide variety of empirical systems and models, collective behaviour has been shown to enhance access to ephemeral patches of resources, reduce the risk of predation and reduce vulnerability to environmental fluctuation. Evolution of collective behaviour should accordingly depend on the advantages of collective behaviour weighed against the costs experienced at the individual level. As an illustrative case study, we consider the potential trade-offs on Malthusian fitness associated with patterns of group formation and movement by migratory Thomson's gazelles in the Serengeti ecosystem.

This article is part of the theme issue ‘Collective movement ecology’.

## 1. Introduction

The past 20 years have witnessed an explosion of interest in the mechanisms contributing to patterns of spatial aggregation and coordinated movement of animal groups [1–4]. Just as in other facets of behavioural ecology, however, our understanding of the cumulative effects of collective behaviour on higher-order processes such as ecology and evolution lags far behind [5]. Our goal in this paper is to develop a theoretical framework for understanding the potential dynamical implications of collective herbivore behaviour with respect to trophic interactions (table 1).

**Table 1.** Model parameters.

parameter	biological meaning
*α*	maximum rate of food intake by each individual herbivore
*β*	plant biomass at which intake is half the maximum value
*Φ*(*V*)	digestible energy content of ingested food
*Ψ*(*V*)	linear function depicting decline in energy intake rates with plant abundance
*Λ*	converts digestible food intake into offspring production for each herbivore
*Γ*	*per capita* risk of mortality in the absence of predation
*a*	area searched per unit time by predator
*b*	handling time for each successful predator attack
*p*	probability of successful prey capture per attack
*c*	impact of predator interference on the rate of attack
*e*	improvement of attack success with increasing vegetation abundance
*G*	group size
*v*	probability that a single herbivore is vigilant
*d*	reduced *p*(successful attack) per individual herbivore group member
*ζ*	probability of fission by herbivore group
*κ*	probability of fusion by two groups
*A*	population range area
*σ*	standard deviation in grass biomass experienced by foragers

Collective behaviours may influence individual fitness through their effects on rates of net energy gain or predation risk [1,6–10]. There is accordingly considerable potential for individuals to improve their individual fitness at the cost of that of other group members by abandoning small groups to join larger groups or pretending to be vigilant while foraging to more effectively compete with conspecifics [11,12]. As more and more individuals choose to closely group, however, individual fitness would be compromised due to increased competition and interference [13]. We currently have little idea how these fitness trade-offs influence the behavioural choices made by individuals within the population, but recent theoretical advances suggest that there is exciting future opportunity to consider how collective behaviour might inform evolutionary outcomes [14,15]. In other words, natural selection acting on individual traits might well be mediated through traits expressed at the group level.

Given that collective behaviour is an emergent property of an assemblage of individuals each with a unique history of experience, genetic composition and set of motivations, one cannot hope at this stage to develop a fully articulated model. Deep understanding of the dynamical properties at the population level will no doubt require highly detailed agent-based models far beyond the capacity of this paper. Since this field is far too young to have developed a deep body of observational data or controlled experiments, here we use conventional consumer-resource theory applied to the concept of Malthusian (i.e*. per capita*) fitness as a lens to identify potentially important linkages that may be worthy candidates for deeper study. Such a framework may be useful in future modelling efforts to consider the potential impact of behavioural variation among individuals needed to assess frequency-dependent selection and the identification of evolutionarily stable strategies.

**Figure 1.**
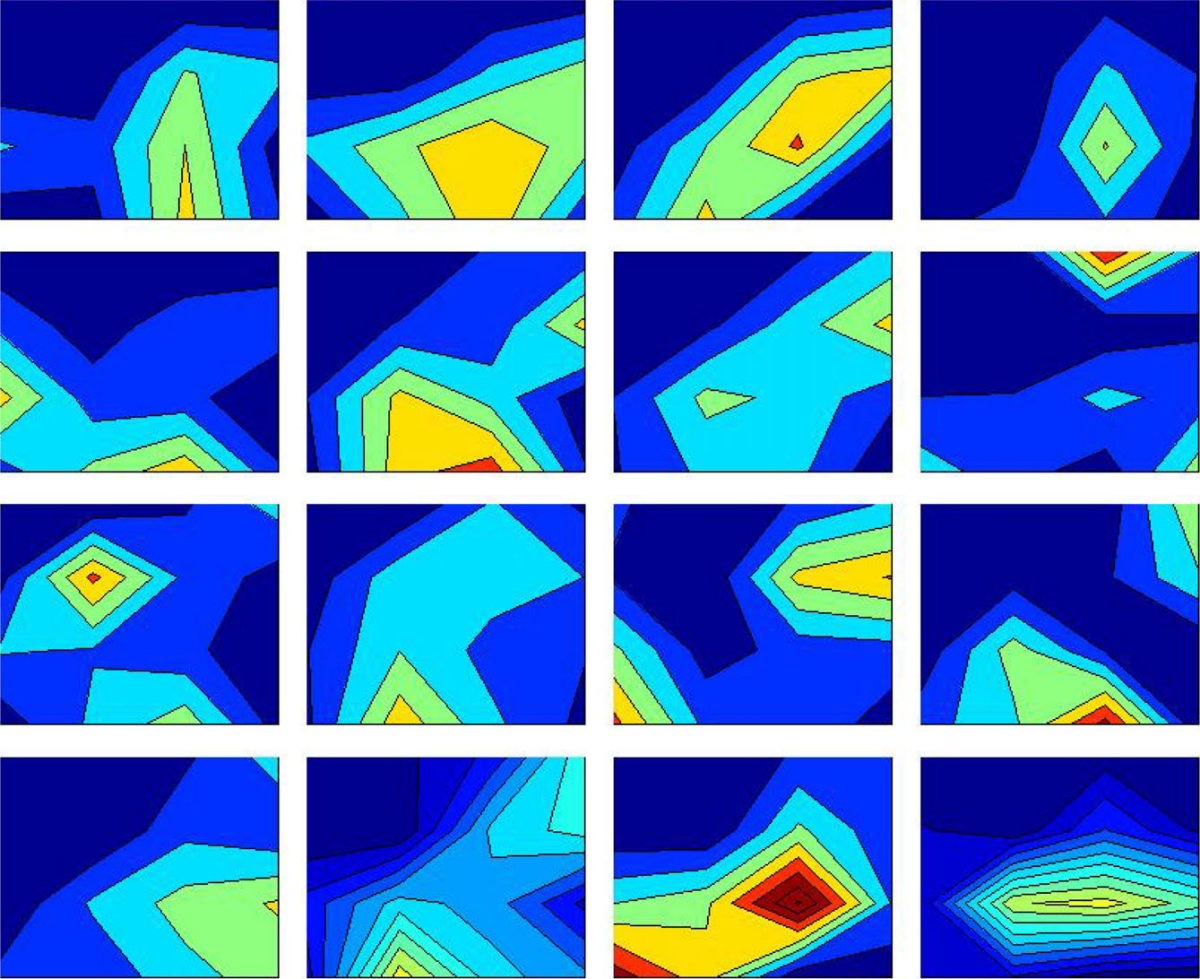
Spatial distribution of Thomson's gazelles across a 4000 km^2^ area of the Serengeti Plains on 16 dates during the wet seasons of 1994 and 1995, measured at two-week intervals. Each panel represents a different census date. Relative abundance in a given census is indicated by shading, ranging from 0 at the dark blue end of the colour spectrum to 300 individuals per km^2^ at the dark red end of the colour spectrum. Figure redrawn from Fryxell *et al*. [17]. (Online version in colour.)

As an empirical example, we apply this modelling framework to the spatial ecology of Thomson's gazelles living in Serengeti National Park, a highly social grazing species that has been the subject of substantial field studies on foraging ecology, space use and predation risk (e.g. [16–19]. Thomson's gazelles migrate seasonally between arid grasslands on the Serengeti Plains used during the wettest part of the year and open savannas in higher rainfall areas in the western corridor and central areas of the park during the dry season [20,21], but are highly nomadic within their seasonal range (figure 1). Social grouping patterns are highly fluid over time, with local aggregations merging and splitting continually from hour to hour, often termed a fission–fusion process [22,23]. Key parameters are available on plant growth dynamics [24], gazelle foraging ecology [16], predation risk [19] and patterns of spatial movement [17,18], which we use to inform our models.

Like many other mobile organisms [23,25], it may be useful to think of gazelle space use at multiple spatio-temporal scales, as a means of breaking down spatial biological complexity into manageable conceptual units. Daily patterns of movement and temporary herd formation at local (less than 1 km) and short-term (hourly) scales are nested within a nomadic pattern of population flux at a regional (10–20 km) medium-term (weekly) scale, which is nested in turn within a migratory circuit between seasonal ranges separated by 50–200 km that is completed on an annual cycle.

We start with mechanistic consideration of how forage abundance and maturational changes in forage nutritional quality serve as fundamental constraints that structure gazelle space use at a fine spatio-temporal scale. We apply this local perspective to evaluate the multiple effects of group formation on the probability of encounter by predators, the probability of prey capture once encountered by predators and interference among prey herd members. We then go on to consider collective behavioural effects at a coarser spatio-temporal scale, through review of a body of theory about the impact of group formation on the effectiveness of herbivore seasonal migration and nomadic movement within seasons. Finally, we integrate across group sizes and levels of forage abundance to consider how local levels of Malthusian fitness might translate into aggregate demographic rates for the entire population.

## 2. Gazelle fitness in relation to forage plant abundance

A key difference between plants and animals as a source of food is that the nutritional quality of plants often declines with maturation, due to the accumulation of structural tissues such as lignin and cellulose that have poor nutritional value. On the other hand, feeding rates of virtually all herbivores increase with plant abundance. Multiplying the rate of forage intake (a function of plant abundance with a positive slope) obtained by feeding on a given plant patch by that patch's nutritional quality (a negatively sloped function of plant abundance) often leads to a hump-shaped (concave down) relationship between herbivore energetic gain and plant abundance (sometimes termed a type-4 functional response).

**Figure 2.**
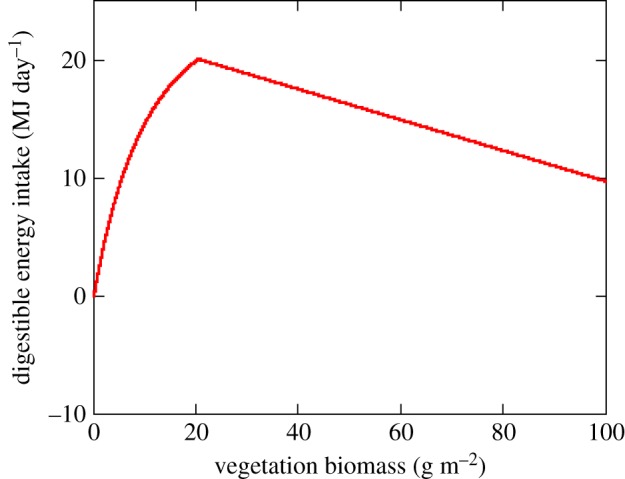
Estimated energy intake (in MJ day^−1^) by Thomson's gazelles in relation to grass biomass (*V*, measured in g dry mass per m^2^) based on equation (2.1) (data from Fryxell *et al*. [17]) The following parameter values were used: *α* = 3420, *β =* 15, *Γ* = 0.0013, *Λ* = 0.00013, *φ*(*V*) = 22.7–0.13 *V*, *Ψ*(*V*) = 0.0112–0.00005 *V*. (Online version in colour.)

Such a hump-shaped relationship is well-demonstrated by Thomson's gazelles (figure 2). Controlled experimental foraging trials conducted on captive animals presented with forage of given maturation stage [16] suggest that rates of food intake (*h*) initially increase with plant abundance (*V*), peak, and then decline at high levels of plant abundance, with the right-hand descending limb a consequence of declining nutritional quality as forage biomass increases, which in turn limits the passage of ingested food (*ψ*(*V*)) through the digestive tract [16,26]2.1
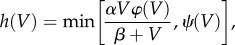
where *α* is the maximum rate of food intake by each individual herbivore, *β* is the plant biomass at which intake is half the maximum value, and *φ*(*V*) is the digestible energy content of ingested food, linearly related to vegetation abundance. *ψ*(*V*) represents a linear function depicting how maximum energy intake declines with plant abundance due to limits on passage time through the digestive tract, typical of many grazing herbivores [26]. This decline in nutritional quality results in energy intake and therefore Malthusian fitness being maximized at low to intermediate levels of plant biomass of approximately 20 g m^−2^ (figure 2), reflecting a trade-off between acquiring and ingesting forage and extracting nutrients from the forage that has been ingested [16]. The highest local concentration of Thomson's gazelles recorded during grassland surveys also occurs in grassland patches whose biomass is roughly 20 g m^−2^ [17], identical with the levels of forage abundance that should maximize energetic gain according to the type-4 functional response model [16]. This provides strong support for the hypothesis that there is a fundamental fitness trade-off between food abundance and food nutritional quality for terrestrial herbivores [26].

If one accepts the common premise that net energy availability translates directly into secondary production, then a similar hump-shaped relationship (often termed the numerical response) should be expected for the Malthusian fitness of gazelles in relation to variation in plant abundance [18]2.2
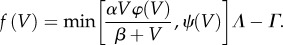
The parameter *Λ* converts digestible food intake into offspring production for each herbivore and the parameter *Γ* represents *per capita* risk of mortality in the absence of predation. As a result of this fundamental trade-off between intake and nutritional quality, Malthusian fitness of gazelles would be expected to be highest at low to intermediate levels of forage abundance, not in patches with high plant abundance.

## 3. Malthusian fitness in relation to energetic gain versus predation risk

All herbivores are faced with the challenging task of securing enough nutrients from a highly dilute resource base to sustain their own reproduction, while avoiding being eaten by a voracious suite of potential predators. We can accommodate this tension, by expanding our Malthusian fitness framework according to the family of tri-trophic consumer-resource models initially developed by MacArthur & Rosenzweig [27], Beddington [28], DeAngelis [29] and Hastings & Powell [30] with the additional wrinkles we have already discussed to accommodate constraints on herbivory imposed by variation in plant nutritional quality [18].

*Per capita* predation risk to each herbivore can be assessed by multiplying the carnivore functional response by carnivore population density (*P*). We base our model on parameter estimates for lions derived from previous behavioural field studies, summarized in Fryxell *et al*. [19]3.1
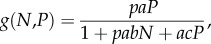
where *a* is the area searched per unit time by carnivores, *b* is the handling time for each herbivore attacked, *p* is the probability of successful attack once prey have been encountered and *c* is the time wasted per encounter between consumers, which scales the impact of predator interference on the overall rate of consumption [28,29,31]. Malthusian fitness for gazelles should accordingly depend on the difference between *per capita* rates of recruitment (*f*[*V*]) versus predation (*g*[*N*, *P*])3.2
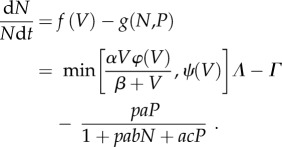


In systems with ambush predators like lions, leopards and hyenas it is conceivable that vegetation abundance also influences predation risk through the effect of heavy vegetation cover on predator visibility. Such effects on attack success are well-documented in lions [32] and seemingly offer a cogent explanation for the preponderance of lions kills in areas of dense vegetation cover near water courses [33] and the avoidance of such thickets by several herbivore species in Serengeti [34]. This could be represented through a positive effect of vegetation abundance on the probability of successful attack (success = *p*[1 − exp(−*eV*)]3.3
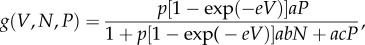
where *e* is the exponential rate of improvement in attack success with each unit increase in vegetation abundance *V*. The indirect facilitation of predators afforded by tall vegetation can induce important changes in the outcome of food chain interactions, ranging from the creation of critical threshold dynamics that result in multiple stable states of predators, herbivores and vegetation to highly complex cycles of abundance [35]. This remains a little-studied topic, but nonetheless one of considerable potential importance.

## 4. Group size effects on predation risk versus energetic gain

We now consider how collective behaviour and the resulting patterns of group formation can influence Malthusian fitness, through modifications in the *per capita* gain (*f*) and cost (*g*) functions described above. We start with a consideration of predation risk, long-recognized as a potential benefit of grouping [11,36]. If the entire population of prey breaks up into tightly knit groups of size *G* that are no more visible to predators than individuals, then the rate of prey encounter = *aN/G*, rather than *aN*, due to the fact that clumps of prey will be encountered far less frequently than the same prey population distributed randomly across the landscape [19,37]. If the predator can at most attack and eat a single prey item, the risk for each individual in a group drops geometrically with group size (1/*G*), due to simple dilution of risk [36]. The combination of both group-dependent effects implies that *per capita* predation risk would be modified in the following manner [19]:4.1
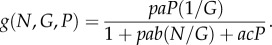


**Figure 3.**
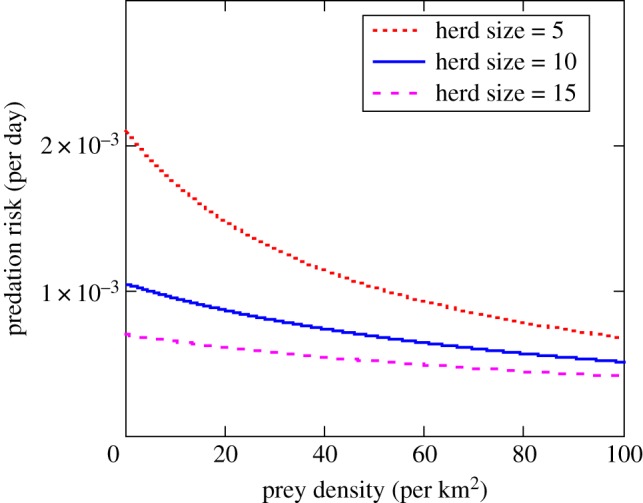
*Per capita* risk of predation by Thomson's gazelles in relation to group size and population density estimated according to equation (3.2). The following parameter values were used for the model: *a* = 4.0, *s* = 0.263, *h* = 0.1, *p* = 0.01. (Online version in colour.)

As a result of decreased search efficiency (equation (4.1)), *per capita* risk of predation would be expected to decline with both gazelle population abundance and group size (figure 3). Given that tight spatial grouping by social herbivores creates potential for foraging interference among group members, there are likely to be costs associated with collective behaviour. For example, many studies of foraging suggest that food intake can be depressed through interference with other group members, either via agonistic interactions or scramble competition for resources [28,29]. Such effects can be accommodated in the gain function *f* by including a herbivore group-dependent interference term *c*(*G*-1) in the denominator of the herbivore functional response4.2
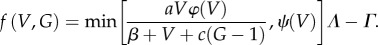

For many herbivores, the probability to successfully evade an attack by a predator would also be expected to depend on prey group size *G*. This benefit can arise in multiple ways. Predators may be less able to successfully attack a group if some group members are vigilant, in which case the probability of attack (*p*) would be expected to decline with the proportion of time each individual is typically vigilant (1−*v*) raised to the power of *G*, the power term being based on the assumption that there is little coordination in vigilance behaviour, where (1−*v*)*^G^* is the Bernoulli trial probability that none of the *G* herd members are vigilant4.3
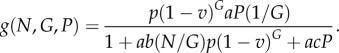
Vigilance also influences energy intake by reducing the time devoted by each individual herbivore to foraging. This can be accommodated in the gain function *f* by including a term for the proportion of time available for foraging (1 *−* *v*) in the numerator of the herbivore functional response4.4
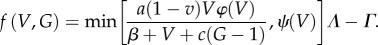


**Figure 4.**
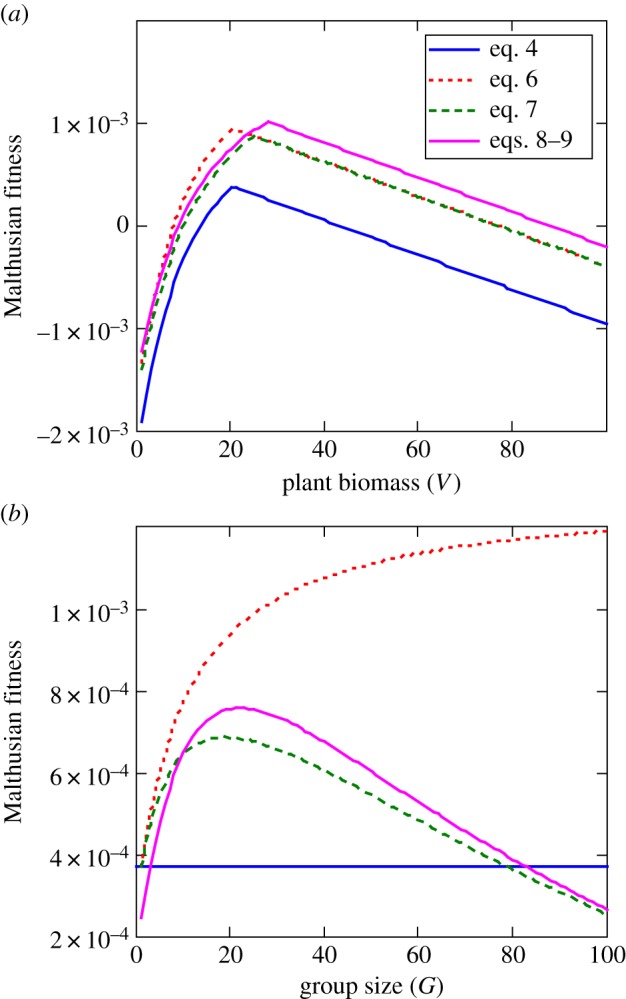
(*a*) Malthusian fitness of Thomson's gazelles in relation to variation in vegetation abundance *V* while holding herbivore group size constant at *G* = 20. Malthusian fitness varies as a function of prey group size-dependent effects on predator search efficiency (equation (4.1)), interference among herbivorous individuals within the prey herd (equation (4.2)), and prey vigilance (equations (4.3) and (4.4)) relative to baseline values (equation (3.2)). (*b*) Malthusian fitness of Thomson's gazelles in relation to variation in herbivore group size *G* while holding vegetation abundance constant at *V* = 20. The following parameter values were used: *a* = 4.0, *s* = 0.263, *h* = 0.1, *v =* 0.05, *γ* = 0.2, *N* = 100, *p* = 0.01, *α* = 3,420, *β =* 15, *Γ* = 0.0013, *Λ* = 0.00014, *φ*(*V*) = 22.7–0.13 *V* and *Ψ*(*V*) = 0.0112–0.00005 *V*. (Online version in colour.)

Attack success once the group has been encountered could also be influenced through collective behaviour through improvement in escape manoeuvres by fleeing group members. Collective information sharing about imminent predation risk can rapidly spread through a close-knit group, allowing remarkably well-choreographed escape behaviour, seen for example in the escape tactics by schooling fish or bird flocks [8,38]. If one assumes that the probability of successful attack due to coordinated escape behaviour or predator confusion decays from a maximum value of *p* with increasing prey group size *G* at a *per capita* rate *d,* then success = *p* exp(*−dG*) and the generic predation risk formula could be altered accordingly4.5
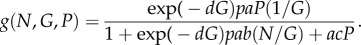
Malthusian fitness is predicted to vary (figure 4) as a result of group-dependent effects on search efficiency (equation (4.1)), herbivore interference (equation (4.2)), predator detection (equation (4.3)) or probability of success per attack (equation (4.4)). Similar results have been found in several previous model variants in the published literature [11,39].

**Figure 5.**
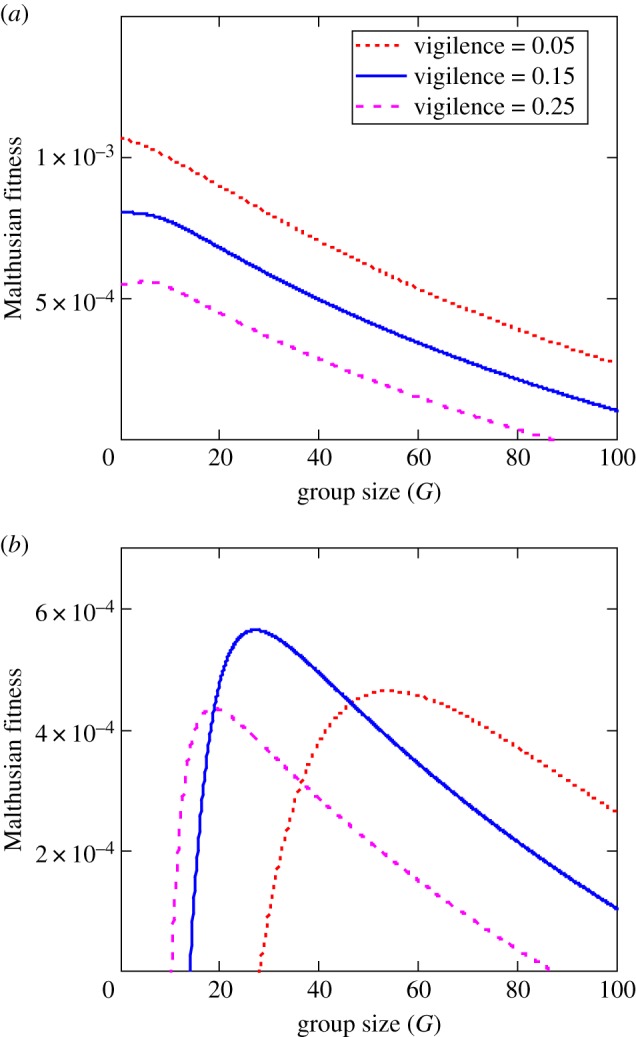
Malthusian fitness of Thomson's gazelles based on trade-offs between *per capita* recruitment and *per capita* predation risk in relation to vigilance level and group size using equation (4.1) in ecosystems with (*a*) low density of predators (*p* = 0.01) and (*b*) high density of predators (*p* = 0.1). Parameter values as in figure 4*a*. (Online version in colour.)

When we combine the group-dependent interference in the modified gain function with the group-dependent benefits in the modified risk function, the net effect typically yields a hump-shaped (concave down) Malthusian fitness function in relation to variation in vegetation abundance (figure 4*a*) and herbivore group size (figure 4*b*). Models based on these trade-offs suggest that the evolutionarily advantageous grouping patterns and levels of vigilance depend on local variation in predation risk, with smaller, poorly vigilant groups favoured in landscape regions with low predator densities (figure 5*a*), and larger, more vigilant groups favoured in landscape regions with high predation risk (figure 5*b*).

## 5. Gazelle spatial dynamics in relation to food resources

Censuses across a 4000 km^2^ portion of the Serengeti plains over 2 years [17] suggest that at an intermediate spatial scale (in the order of tens of kilometres over monthly intervals) gazelle populations move in a nomadic fashion, seemingly in response to local variation in rainfall and its effects on food availability (figure 1). The foraging models outlined earlier suggest that gazelle energy gain is highest when feeding on grass swards that are sparse, on the order of 20 g m^−2^, a level roughly equivalent to the biomass of a mown lawn [17]. Although gazelle spatial distribution is highly variable from month to month, much of the variation in both space and time can be explained by adaptive movement responses, as gazelles demonstrably concentrate in local grassland patches with intermediate levels of vegetation abundance [17]. This finding suggests that while not all gazelles succeed in locating the optimal patches, on average the attractiveness of neighbouring patches to a focal individual is proportionate to energetic value. Stochastic simulations suggest that adaptive nomadic movement is essential for modelled gazelles to persist in the face of stochastic variation in plant growth rates and that unrestricted access to a large landscape has important bearing on the probability of population persistence [18]. This is probably particularly crucial in semi-arid environments, where spatial and temporal variation in rainfall is often pronounced. This process bears strong similarity to the portfolio effect of spatial variation in fitness that has been argued for assemblages of salmon runs within and among river systems [40,41].

Despite their utility, current models and supporting field studies offer little insight, however, into the precise behavioural mechanism by which Thomson's gazelles or wildebeest track the shifting mosaic of food patches over time and space. It seems unlikely that any single individual can reliably sample such a large landscape effectively and thereby make informed patch choice decisions at the landscape scale. Both theoretical models [42] and experiments with schooling fish [43] suggest that individuals in large groups may be better able to sense and respond to rapidly shifting resource availability in efficient manner. Such improved sensing of and response to resource gradients may be the result of one or more of the following mechanisms [4]: (i) many individuals pooling noisy estimates of the gradient (many wrongs); (ii) a subset of better-informed individuals leading entire groups (leadership) and (iii) the group acting as a distributed sensory array able to compare vegetation quality at spatial scales much longer than would be possible for any individual (emergent sensing).

**Figure 6.**
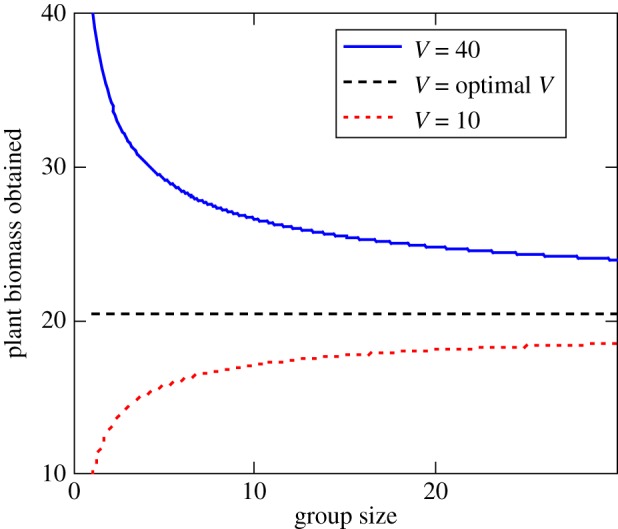
Vegetation biomass obtained by foragers when tracking of the environmental resource gradient is enhanced by collective behaviour (equation (6.2)). Three scenarios are shown: *V* = 10 g m^−2^, *V* = the energy-maximizing level of 20.3 g m^−2^ and *V* = 40 g m^−2^. (Online version in colour.)

**Figure 7.**
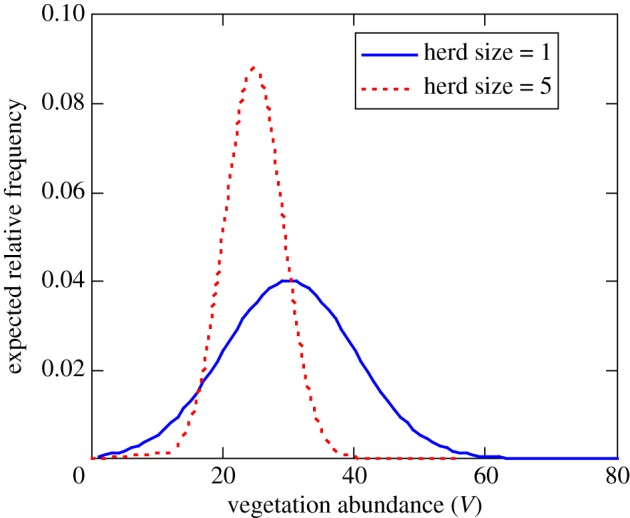
Expected distribution of vegetation levels experienced by animals foraging alone or in groups of 5 across an heterogeneous landscape as predicted by equations (4.5) and (5.1), with vegetation abundance distributed normally with *μ* = 30 and *σ* = 10. (Online version in colour.)

Models of mobile populations collectively sensing environmental gradients associated with patchy resources suggest the use of such collective behaviour could greatly affect both population and migratory dynamics [44]. Here we attempt to incorporate such spatial effects into our mean-field model by adjusting the range of variation in forage abundance sampled by groups of size *G* from a distribution of vegetation patches, whose abundance is distributed normally with mean *μ* and standard deviation *σ*
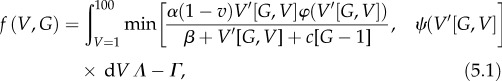
with5.2
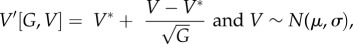
where V* is the vegetation level that optimizes energy intake (i.e. the peak of figure 2). This formulation assumes that larger groups are better able to search out, or bias their movement towards, areas with better forage opportunities (figure 6). When *G =* 1, the individual samples vegetation abundance randomly from the heterogeneous distribution that occurs across the landscape. Groups sample vegetation abundance from a narrower distribution, whose mean approaches the optimum as 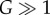 (figure 7). This is phenomenological, rather than based on a specific mechanism; however, it is consistent with collective searching due to many wrongs and emergent sensing. Many wrongs predict that error should decrease with group size as 1/√*G* [4]. In the case of emergent sensing, the group's ability to detect a gradient should be proportional to the length scale of the group (√*G*, assuming a *roughly* circular group), so it is also reasonable to assume that error drops off as 1/√*G*. Seasonal variation in gazelle distribution at coarse temporal and spatial scales strongly suggests that movement flux across the savannah landscape is shaped by the need to make efficient use of ephemeral and spatially unpredictable food resources. Here we have modified the foraging term to incorporate theory suggesting that collective behaviour should improve the capacity of gazelles to locate suitable forage and thus meet their energetic needs across this shifting mosaic, while incorporating costs due to intra-group competitive interactions.

## 6. Group formation as a fission–fusion process

**Figure 8.**
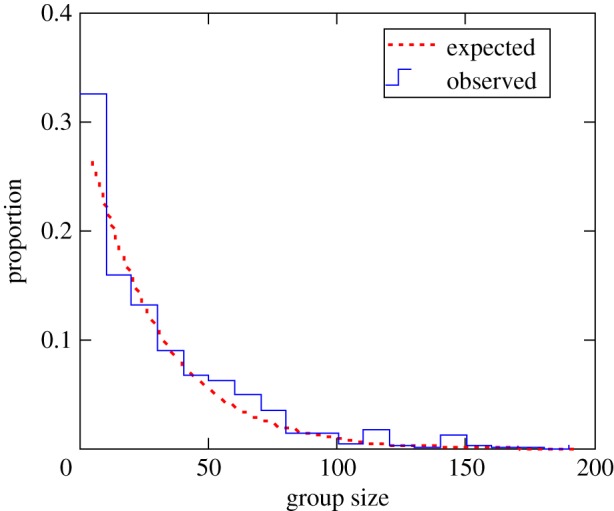
Observed group size distribution for the Serengeti population of Thomson's gazelles estimated over 60 censuses (2004–2009) using the methodology described in Fryxell *et al*. [19]. The observed distribution is well-approximated by an exponential curve (*y* = 0.25exp(−0.0029*x*), *p* < 0.001, *R*^2^ = 0.85) fit to binned data. (Online version in colour.)

The frequency distribution of group sizes in Thomson gazelles is well-approximated by negative exponential or power functions in Thomson's gazelles (figure 8) [19] and a variety of other taxa [45–48]. Such grouping patterns can be readily explained by coalescence models originally applied to the formation of polymers and other long-chain molecules. The logic behind these models can be remarkably simple: individuals have fixed probability of joining other individuals whenever encountered, thereby forming loose temporary groups, but groups similarly have a finite probability of budding off into fragments of random size. For example, Gueron & Levin [49] and Gueron [50] demonstrated that if the probability of fission *q* scales linearly with size of the group (*G*) such that *q*(*G*) = *ζG* and that groups fuse with constant probability *κ* whenever they meet, then over time this mix of fission and fusion events will result in an exponential probability distribution of group sizes6.1
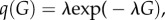
where *λ* satisfies the following relationship:6.2
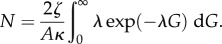

So long as the probabilities of joining groups or group fragmentation are constant or positively related to group size, repetition of the episodes of fission–fusion over time leads to an equilibrium frequency distribution similar to that shown by Thomson's gazelles (figure 8).

A variety of models of group formation have been developed using similar assumptions [47,48], generally yielding either exponential- or power-scaled distributions of group size. Data consistent with these qualitative predictions are well-documented in a number of published studies of large mammalian herbivores [10,45,46], fish [47] and even invertebrates [48], suggesting that simple processes of fission and fusion offer a robust and potentially useful way to think about group-dependent interactions and expectations about group size distributions.

## 7. Group size, population abundance and population rate of increase

**Figure 9.**
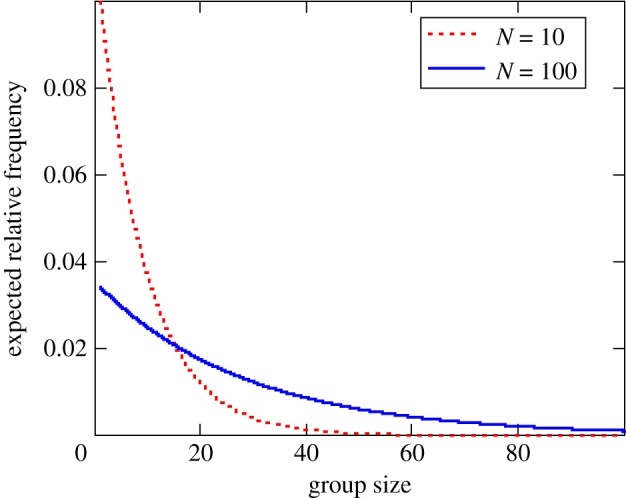
Distribution of group sizes predicted by Gueron & Levin's [50] fission–fusion model with the probability of fission (*q*(*G*) = *ζG, ζ* = 1) a positive linear function of group size and a constant probability of fusion (*κ* = 0.004) when groups encounter each other at low (*N* = 10 individuals per km^2^) and high (*N* = 100) population densities, spread across an area *A* = 4000 km^2^. (Online version in colour.)

A common property of such fission–fusion models is that the number and size of groups depend on population abundance (figure 9). As a result, rates of population change will be shaped not only by the relative abundance of predators and prey, but also the distribution of group sizes in the prey population. The aggregate effect on population vital rates can be estimated by integrating Malthusian fitness predicted by our group size-dependent models across the predicted group size distribution (figure 8)7.1
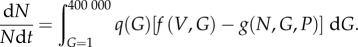

Here we are integrating *G* from 1 to a maximum of 400 000 animals (the total population size for Thomson's gazelle in Serengeti).

**Figure 10.**
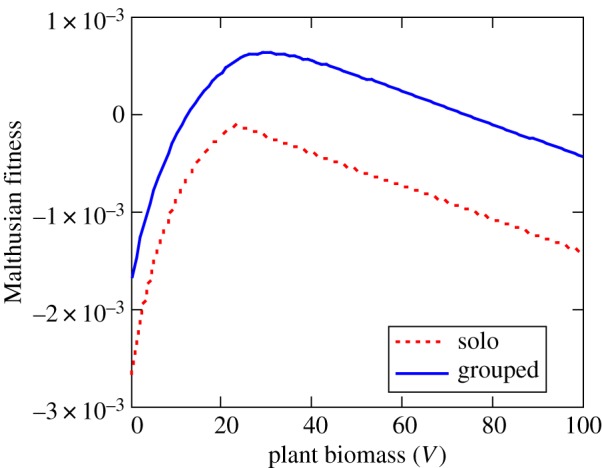
Malthusian fitness by Thomson's gazelles based on trade-offs between *per capita* recruitment and *per capita* predation risk integrated over the range of group sizes predicted by Gueron & Levin's [49] fission–fusion model for low population density (*N* = 10 individuals per km^2^) and high population density (*N* = 100) as estimated by equation (6.2). Other parameters were as follows: *A* = 4000, *α* = 3420, *β* = 15, *γ* = 0.2, *φ*(*V*) = 22.7–0.13 *V*, *Ψ*(*V*) = 0.0112–0.00005 *V*, *p* = 0.001, *v* = 0.05, *a* = 4.0, *s* = 0.263 and *h* = 0.1. (Online version in colour.)

Choosing values for *ζ*, *κ* and *λ* consistent with the observed frequency distribution of group sizes and an average population density of *N* = 100 individuals per km^2^ spread across an area *A* = 4000 km^2^ of the Serengeti Plains (yielding a total population of 400 000 gazelles), we can apply this model to predict the impact of changing population abundance of Thomson's gazelles in the Serengeti ecosystem. If one assumes that gazelles do not change their rates of fission and/or fusion with changing levels of population density, then at high population densities the mean size of gazelle groups would be expected to increase, albeit with a flattened distribution relative to the sharply declining exponential distribution expected from a much smaller population (figure 9). We can then link models for Malthusian fitness with the size distribution models. For example, we can imbed the Malthusian fitness relationships from equations (4.3) and (4.4), which assumes group-dependent effects on prey encounter rates, prey interference and prey vigilance into the simple fission–fusion model in equation (6.1). Under these conditions, predation risk is predicted to be substantially increased when prey densities are low than when prey densities are higher, resulting in a dramatically altered rate of population growth (figure 10), even when food resources are optimal. If grouping patterns are compromised severely, this inversely density-dependent process suggests that predation risk might be increased sufficiently to induce an Allee effect leading to inevitable collapse once population abundance falls below a critical threshold.

## 8. Ecological and conservation implications of collective behaviours

The simple models we have outlined here clearly suggest that the processes contributing to movement, spatial heterogeneity, group formation and fragmentation have noteworthy consequences for large herbivore populations striving to balance challenges in resource acquisition against the risks of predation. Joining together with others can substantially reduce the risk of predation while individual herbivores are preoccupied with forage acquisition. Group-forming foragers are also quite probably much more adept at locating ephemeral patches of food, taking advantage of the many wrongs principle or emergent sensing to efficiently sample large landscapes with food patches generated by unpredictable and highly localized rainfall.

On the other hand, time devoted to vigilance, direct interference among nearby foragers, and local depression of resource supplies while foraging in the kinds of large groups often seen in Serengeti could plausibly impose fitness costs of considerable magnitude. Given this trade-off, seeking out locations with lower densities of predators or other competitors or concentrating in habitat types that reduce the probability of successful attack may enhance fitness just as much as grouping tightly together. Small wonder, perhaps, that grouping patterns and the spatial distribution of Serengeti herbivores are continually shifting over time and space. The range of alternative behavioural strategies yielding comparable fitness may mitigate against stable group formation, particularly in migratory species like Thomson's gazelles.

Spatio-temporal variation in fitness recurs at multiple scales [25]. Broad seasonal patterns of migration across the Serengeti landscape in relation to monsoonal transitions in rainfall intensity from the Serengeti Plains to the margins of Lake Victoria generate an important source of fitness variation which gazelles, zebra and wildebeest strongly respond in predictable fashion. At a slightly finer scale, regional variation in rainfall compounded with consumption by herbivores, generates spatial fitness heterogeneity to which gazelles strongly respond. Finally, at fine spatio-temporal scales there is significant opportunity for fitness enhancement available through group formation. By integrating these sources of variation it should be possible, in principle, to predict the aggregate effects of spatial movement processes operating at multiple scales [23]. Here we demonstrate that it is similarly possible to integrate variation in resource levels across a heterogeneous vegetation landscape and group sizes across the entire consumer population to better understand temporal variation in vital rates. Field measurement of these same effects obviously presents an enormous logistical challenge. But without a detailed accounting of scale-dependent effects, it is hard to imagine achieving a robust and reliable understanding of the scale-dependent processes that shape population processes in such spatially extended ecosystems [25].

The importance of nomadic and collective movement in the Serengeti extends beyond gazelles. Modelling of migratory Serengeti wildebeest herds similarly demonstrated that persistence of a million wildebeest depends strongly on unrestricted access to large expanses of savannah grasslands [51]. Creation of movement barriers, such as high-speed road networks would almost certainly reduce the carrying capacity for wildebeest as well as increasing year-to-year variation in abundance [52]. Given that savannah ecosystems like Serengeti are particularly prone to wide swings in rainfall from year-to-year and from one spatial location to the next, these thought models suggest that mobility may be an essential life-history trait in Thomson's gazelles, wildebeest and perhaps many other large herbivores living in highly stochastic environments.

A common and intuitive result is that reliance on beneficial social behaviour could result in Allee effects [44,53–55]. In the case of collectively navigating populations, population density can play a critical role in ensuring sufficiently efficient information transfer to guide migratory or nomadic movements. If reduction in population size leads to smaller group sizes on average, as predicted by simple fission–fusion models (figure 9), then the benefits that population receives from collective behaviour will be diminished, which may, in turn, lead to further population declines (figure 10). Such positive feedback would lead to a critical population size, below which the collective navigation becomes ineffective and the population is less able to track resources or complete a migration, to a point where continued persistence of the migratory population itself is threatened [44,54]. Models used to manage populations that do not take such social effects into consideration would not predict population collapse [44]. Our tri-trophic models similarly suggest that solitary gazelles would be at considerably risk of extinction by predator populations at high density, whereas group-forming individuals can greatly reduce this risk through a variety of behavioural mechanisms. This suggests that there may be appreciable conservation benefits from improved understanding of Allee effects arising from critical transitions (tipping points) due to collective behaviour.

While we have no ironclad proof that the ebb of flow of herbivores across every ecosystem is vital to sustaining ecological processes, tantalizing hints emerge from the theoretical literature. A variety of consumer-resource models suggest that asynchronous movement leading to substantial spatial heterogeneity in recruitment patterns by consumers and their resources should tend to dampen the amplitude of population fluctuations, thereby improving population persistence [56–59]. If collective behaviour contributing to more efficient resource use leads to increased spatial heterogeneity in herbivore abundance, then collective behaviour may well dampen the intensity of trophic interactions with their predators as well.

Ironically, the capacity for collective behaviour to rapidly identify new foraging sites can itself prove problematic, if these novel foraging sites turn out to be in risky habitats due to anthropogenic changes that the groups cannot or do not detect. For example, Canadian bison herds in Prince Albert National Park that learned about new foraging sites via collective behaviour were exposed to hunting pressure that resulted in rapid population collapse [60]. Such an ecological trap is all too likely when habitat-mediated fitness attributes are altered by human disturbance across landscapes [61]. Evolutionarily stable behavioural responses may be tenable no longer in a rapidly changing world. The effectiveness of spatial collective behaviour will also break down of course when anthropogenic barriers are erected for the simple reason that gradient-climbing processes require landscape continuity. A recent meta-analysis across 57 different species demonstrated that movement rates by terrestrial mammals have declined substantially in landscapes that are heavily disturbed by human activities [62]. Hence roads, fences, pipelines and other linear features may represent conservation threats of surprising magnitude [63].

The obvious reliance of so many Serengeti herbivores on various forms of collective behaviour suggests that social behaviour itself is a life-history trait of vital importance. Moreover, the emerging consensus that there may be threshold levels of abundance below which adaptive collective behaviours such as efficient resource tracking and predator avoidance break down suggests that loss of collective behaviour can itself generate Allee effects [44,54]. Both features argue that management and conservation policies are needed that guard against the loss of adaptive collective behaviour, such as through imposition of fences or major road networks, or tourist disturbance.

## Data accessibility

This article has no additional data.
